# A phase 1 study of ASTX727 plus talazoparib in patients with triple‐negative or hormone resistant/HER2‐negative metastatic breast cancer

**DOI:** 10.1002/cncr.70407

**Published:** 2026-04-14

**Authors:** Kathy D. Miller, Alexandra Thomas, Sandra Althouse, Yong Zang, Erin Conder, Ryan Burgos, Bryan P. Schneider, Tarah Ballinger, Emily Douglas, Katherine Ansley, H. Josh Jang, Woonbok Chung, Jean‐Pierre Issa, Kenneth P. Nephew, Feyruz V. Rassool

**Affiliations:** ^1^ Indiana University Melvin and Bren Simon Comprehensive Cancer Center Indianapolis Indiana USA; ^2^ Duke Cancer Institute Duke University Durham North Carolina USA; ^3^ Biostatistics and Health Data Science Indiana University School of Medicine Indianapolis Indiana USA; ^4^ Van Andel Institute Grand Rapids Michigan USA; ^5^ Wake Forest University School of Medicine Winston‐Salem North Carolina USA; ^6^ Coriell Institute for Medical Research Camden New Jersey USA; ^7^ University of Maryland School of Medicine Baltimore Maryland USA

**Keywords:** breast cancer, DNA methyltransferase I, epigenetic, homologous recombination DNA repair, inflammasome, PARP inhibitor, phase 1 clinical trial

## Abstract

**Background:**

Poly(adenosine diphosphate ribose) polymerase (PARP) is recruited to DNA damage sites along with epigenetic factors such as DNA methyltransferase 1 (DNMT1). Inhibitors of DNMT modulate reactive oxygen species (ROS)‐cyclic adenosine monophosphate (cAMP)/Protein Kinase A signaling and induce a “BRCAness phenotype” that further sensitizes cells to PARPi. In preclinical studies, combined DNMTi + PARPi therapy was effective in both triple‐negative (TNBC) and hormone resistant (HRBC) models with intact BRCA.

**Methods:**

The authors conducted a phase 1 study combining the oral DNMTi ASTX727 with the PARPi talazoparib in patients with previously treated TNBC or HRBC. Patients with deleterious mutations of BRCA were excluded. A classical 3+3 design guided dose escalation/de‐escalation, and 28 days constituted each cycle. Serial peripheral blood mononuclear cells (PBMCs) were analyzed for changes in methylation using the Infinium Methylation EPIC BeadChip and LINE1 sequencing.

**Results:**

Thirty‐four evaluable patients were enrolled and treated in eight dose cohorts. Myelosuppression was common with grade >3 neutropenia in 42% and grade 3 anemia and thrombocytopenia in 13%. Dose‐limiting toxicity was limited to neutropenia. Efficacy was assessed in 29 patients. There were no objective responses, six patients had stable disease persisting for >4 months in three patients. LINE1 demethylation ranged from ∼2%–10% and immune‐specific CpGs (methylation in immune cells) changed 1%–5% at day 15. Methylation changes were not dose‐dependent.

**Conclusions:**

ASTX727 plus talazoparib produces significant myelosuppression without other adverse events. Modest methylation changes in PBMCs were detected. There were no objective responses, but some heavily pretreated patients had stable disease for >4 months despite the attenuated doses.

## Abstract 2

In the first clinical trial combining talazoparib and ASTX727, myelosuppression limited drug exposure but the combination was otherwise well tolerated; modest methylation changes in peripheral blood mononuclear cells were detected. There were no objective responses, but ∼10% of patients without germline BRCA mutations had stable disease persisting for more than 4 months.

## INTRODUCTION

Triple‐negative breast cancer (TNBC) accounts for approximately 15% of breast cancers. Though immune checkpoint inhibition has a role in some patients,^1^, ^2^, ^3^, ^4^, ^5^ chemotherapy remains the mainstay of treatment. Patients with hormone sensitive disease may benefit from an increasing array of antiestrogen regimens,^6^, ^7^ but chemotherapy is recommended when hormone resistance invariably develops. Though multiple chemotherapy agents are available, responses are often short‐lived and treatment‐related morbidity is substantial. Poly(adenosine diphosphate ribose) polymerase (*PARP*) inhibitors (*PARP*i) have significant activity in patients with metastatic breast cancer associated with a deleterious mutation in *BRCA1* or *BRCA2*.^8^, ^9^, ^10^, ^11^ More recent data expand the use of *PARP*i monotherapy to patients with mutations in some (*PALB2*), but not all (*ATM, CHEK2*), other genes implicated in homologous recombination. Additionally patients with somatic rather than germline *BRCA* mutations may benefit.^11^ Patients with deleterious *BRCA1* mutations frequently develop TNBC, whereas those with mutant *BRCA2* preferentially develop estrogen sensitive disease. Consequently, there was early hope that *PARP*i would be useful in sporadic TNBC and hormone resistant disease (HRBC). Unfortunately, clinical reality quickly dashed those hopes, as activity in patients without defined mutations in *BRCA* or *PALB2* has been minimal.^12^, ^13^

Investigation of the mechanism underlying the cytotoxic effects of *PARP*i have shown that the most potent agents trap PARP at DNA damage sites,^14^, ^15^, ^16^, ^17^ leading to persistent DNA damage and cell death. We have shown that PARP interacts with, and is recruited to, DNA damage sites along with epigenetic factors, such as DNA methyltransferase 1 (DNMT1).^18^ Clinically available PARPis also trap DNMTs into DNA, amplifying the effect. In addition to enzyme trapping, our preclinical data demonstrates that accumulation of reactive oxygen species (ROS) and modulation of the ROS‐cAMP/Protein Kinase A signaling axis by decitabine or guadecitabine significantly enhances the response to talazoparib in TNBC and HRBC cells, resulting in increased sensitivity to talazoparib.^16^, ^18^ More recently, we have shown that the combination of DNMTi and *PARP*i induces ZNFX1, regulating mitochondrial ROS and leading to mitochondrial damage, DNA leak into the cytosol, and activation of STING similar to the response to viral pathogens.^19^ This “pathogen mimicry response” activates inflammasome signaling leading to a “BRCAness phenotype” in models with intact *BRCA*.^20^ Importantly, the synergy was seen regardless of BRCA status and in models with no mutations in genes associated with homologous recombination.^21^, ^22^, ^23^, ^24^, ^25^

Based on our preclinical data, we hypothesized that combining DNMTi + *PARP*i would 1) increase PARP trapping at DNA damage sites, 2) induce ROS, and 3) activate the inflammasome leading to a BRCAness phenotype. We further hypothesized that together these three distinct mechanisms of synergy would lead to cytotoxicity in TNBCs and HRBCs with intact *BRCA*. A phase 1 trial of decitabine plus talazoparib in patients with refractory acute myelogenous leukemia (AML) was ongoing (NCT02878785),^26^ but there was no experience with DNMTi+PARPi combination regimens in patients with solid tumors.

Our preclinical studies were conducted with the parent DNMTi decitabine and its analogue guadecitabine. Decitabine requires intravenous administration due to rapid clearance by cytidine deaminase (CDA) in the gut and liver.^27^ 2′‐Deoxy‐5‐azacytidylyl‐(3′ → 5′)‐2′‐deoxyguanosine sodium salt (guadecitabine) is metabolized to decitabine but is resistant to modification by cytidine deaminase resulting in a longer half‐life.^28^ However, guadecitabine requires subcutaneous administration and its clinical future was uncertain. Consequently, we chose ASTX727, a novel fixed dose combination of decitabine with cedazuridine, a cytidine deaminase inhibitor with excellent oral bioavailability. A phase 1 dose‐finding study found that a fixed dose oral combination of 35 mg of decitabine and 100 mg of cedazuridine produced similar decitabine exposure as decitabine administered intravenously at 20 mg/m^2^ as a 1‐hour infusion. In a confirmatory phase 2 study, ASTX727 (35:100) successfully emulated the AUC exposures and LINE‐1 demethylation of 20 mg/m^2^ intravenous (iv) decitabine in a 5‐consecutive‐day regimen; clinical response and safety data appeared similar to that reported for decitabine 20 mg/m^2^ iv as well.^29^, ^30^ In addition, the day 1, 3, 5 schedule maintained DNMTi. A low dose ASTX727 formulation containing 10 mg decitabine with 100 mg cedazuridine became available during the conduct of this trial, providing greater flexibility in dosing. Given the potential mechanisms of synergy, we selected talazoparib as the *PARP*i for this trial to maximize PARP trapping. Use of ASTX772 with talazoparib allowed us to test our underlying hypothesis with an all‐oral regimen that would be preferred by patients and would facilitate chronic therapy.

## MATERIALS AND METHODS

### Patient eligibility

Patients with histologically or cytologically confirmed TNBC or hormone‐positive, HER2‐negative metastatic breast cancer were eligible. Disease phenotype was defined based on American Society of Clinical Oncology (ASCO)‐College of American Pathologists guidelines except that patients with weak estrogen receptor (ER) and progesterone receptor (PR) staining in <5% of cells were considered TNBC. Patients with TNBC must have had at least one prior chemotherapy regimen for metastatic disease. Patients with HRBC were required to have progressed on endocrine therapy with a CDKi in the metastatic setting; prior chemotherapy was allowed but not required. Patients had to have an Eastern Cooperative Oncology Group (ECOG) performance status of 0–1 with adequate hepatic (total bilirubin < upper limit of normal [ULN] unless documented Gilbert’s disease; aspartate aminotransferase and alanine aminotransferase <3.0 ULN) and hematologic (absolute neutrophils >1500/mm^3^; platelets >100,000/mm^3^; hemoglobin >9.0 g/dL) function. Measurable or evaluable disease was allowed. Patients with active central nervous system (CNS) disease were excluded, but stable CNS involvement (>4 weeks from definitive CNS treatment with stable or decreasing corticosteroid dose) was allowed. Patients with known deleterious mutations of BRCA1, BRCA2, or PALB2 were excluded given the known activity of PARP inhibitors in those patients and our interest in testing synergy in patients without such mutations. The Indiana University and Wake Forest University institutional review boards reviewed and approved the protocol, all subsequent protocol amendments, all serious adverse events, and annual progress reports. All patients provided individual written informed consent before screening and study entry.

### Funding

Research funding provided by the Van Andel Institute through the Van Andel Institute–Stand Up to Cancer Epigenetics Dream Team (principal investigator [PI]: J.‐P.I.) and the Breast Cancer Research Foundation (K.D.M.). Drug supply and partial research support provided by Astex and Pfizer. The funders had no role in study conduct or data analysis. The PI made the decision to publish and drafted the manuscript; all authors provided input and approved the final version. The funders reviewed the draft manuscript before submission but did not provide comments or influence the content.

### Treatment plan

Patients were enrolled and treated with oral ASTX727 and talazoparib in eight successive dose cohorts (Table 1). Both agents were administered orally once daily; days of administration varied based on the cohort. Patients were asked to fast 2 hours before and 2 hours after ASTX727 administration; clear liquids such as water, black coffee, or tea were allowed during the 4‐hour fasting period. Talazoparib was administered without regard to fasting. Patients continued treatment until disease progression or unacceptable toxicity. Prophylactic antiemetics were not recommended but could be added at the investigator’s discretion. Prophylactic use of white or red blood cell growth factors was not allowed but could be added to manage toxicity in accordance with ASCO guidelines, if needed. Concurrent denosumab or bisphosphonates were allowed in patients with bone involvement.

**TABLE 1:** Dose cohorts.

Dose level	Pts	ASTX‐727		Talazoparib		DLTs^a^
		Decitabine:cedazuridine (mg)	Days	Dose (mg)	Days	
1	3	35:100	1–5	0.5	1–28	2
2	3	35:100	1–3	0.5	1–28	2
3	3	35:100	1, 3 ,5, 15, 17, 19	0.5	1–7 and 15–21	3
4	2	35:100	1, 3, 5 15, 17, 19	0.25	1–7 and 15–21	1
5	6	10:100	1, 3, 5	0.5	6–21	0
6	7	10:100	1–5	0.5	6–21	1
7	3	10:100	1, 3, 5	0.25	1–21	0
8	7	10:100	1, 3, 5	0.5	1–21	0

Abbreviations: DLT, dose‐limiting toxicity; Pts, patients.

^a^ All DLTs were myelosuppression, primarily neutropenia.

### Safety and efficacy assessments

Toxicity was assessed based on NCI Common Terminology Criteria for Adverse Events (CTCAE) version 5.0. Patients were evaluated for dose‐limiting toxicity (DLT) defined as Grade 4 neutropenia or thrombocytopenia lasting >7 days, or clinically significant grade >3 nonhematologic toxicity in cycle 1; 28 days constituted each cycle. Based on the mechanism of action, single agent toxicities, and experience combining DNMTis with cytotoxic chemotherapy, myelosuppression was expected. Consequently, a complete blood count was obtained weekly during the first two cycles, on days 1 and 15 of cycle 3, and day 1 of each subsequent cycle. Doses were held or reduced for excess myelosuppression; white blood cell growth factors were not used to maintain dosing. Patients were evaluated clinically, and serum chemistries were obtained on days 1 and 15 of the first three cycles, and day 1 of each subsequent cycle. Disease status was assessed according to the Response Evaluation Criteria for Solid Tumors version 1.1^31^ every two cycles for the first 24 weeks and every three cycles thereafter.

### Changes in peripheral blood mononuclear cell methylation

Peripheral blood mononuclear cells (PBMCs) were collected for assessment of global DNA methylation of LINE‐1 and other selected genes at baseline, before treatment on cycle 1 day 8, cycle 2 day 1, cycle 2 day 8, and then day 1 of cycles 3, 5, and 7. Genome‐wide methylation profiling was conducted using the Infinium Human Methylation EPIC v2.0 Bead Chip platform (Illumina, California, USA). This platform detects methylation levels at over 935,000 CpG sites. Assay process included DNA hybridization to bead arrays, enzymatic extension, and fluorescent staining, enabling precise measurement of methylation at individual sites. We generated DNA methylome data from PBMCs collected at baseline (C1D1), cycle 1 day 15 (C1D15), cycle 2 day 1 (C2D1), and cycle 2 day 15 (C2D15). The raw methylation data were processed with the signal extraction and summarization of array methylation experiments (SeSAMe) R package. This software normalized signal intensities, corrected for dye bias, and filtered low‐quality probes, ensuring the reliability of the resulting β value matrices. These β values ranged from 0 (unmethylated) to 1 (fully methylated). Deconvolution analysis of the methylation data was conducted by HEpiDISH algorithm.

### Bisulfite repetitive element polymerase chain reaction

In addition to methylation profiling, high resolution analysis was performed to assess global DNA methylation changes using our previously published method.^32^ Briefly, genomic DNA was isolated from PBMCs (*n* = 62 samples; corresponding to 21 patients studied on day 1, day 15, and C2D1), bisulfite treated, and polymerase chain reaction (PCR) amplified in nonstringent conditions using PCR primers designed from a consensus LINE repetitive element sequence that allows the amplification of a pool of several thousand repeats. To assess the decrease in global DNA methylation, the sequence difference in this pool of amplified repeats was quantitated using pyrosequencing. In addition, to extend demethylation studies from LINE1 to single loci, bisulfite repetitive element PCR was used to analyze methylation of INS6 (also known as incretin peptide 6), which we have previously shown to have a normally highly methylated promoter CpG island.^32^

### Statistical and bioinformatic analyses

The primary objective of this phase 1 trial was to evaluate the safety and tolerability of ASTX727 in combination with talazoparib in patients with metastatic TNBC or HRBC. Three patients were enrolled into each successive cohort. If none of the three patients in a cohort experienced DLT, accrual to the next cohort commenced. If two or more patients experienced DLT, the previous dose level was considered maximum tolerated dose (MTD). If DLT was observed in one of three patients, three additional patients were to be added to that dose level. If no additional DLTs were reported, accrual to the next cohort commenced. If two or more of the six patients experienced DLT, the previous dose level was considered the MTD. Patients who stopped therapy before completing the DLT evaluation period for reasons other than toxicity were replaced. The secondary end point was to evaluate efficacy; circulating biomarkers of response and biologic activity were exploratory end points. Demographic and other characteristics were summarized as median (range) for continuous variables and number and percentage for categorical variables. Overall response rate (ORR) and clinical benefit response (CBR; complete response/partial response/stable disease (CR/PR/SD) at 18 weeks for TNBC, 24 weeks for HRBC) were determined and the percentage and 95% confidence intervals were calculated, if possible. The Kaplan–Meier method was used to analyze progression‐free survival (PFS) and overall survival (OS). Median with 95% confidence intervals was calculated along with the 6‐month probability. All analyses were performed using SAS Version 9.4 (Cary, North Carolina).

## RESULTS

Thirty‐four patients were enrolled and evaluable for toxicity. Median age was 59 years (33–76). Most patients were White (85%); 12% identified as Black. Three‐quarters (73.5%) of patients had an ECOG performance status of 1 at study entry. Twenty‐nine patients were evaluable for response; one did not have measurable disease at baseline and four stopped treatment without post‐baseline disease assessment. Two stopped therapy due to prolonged myelosuppression and began other anticancer therapy without intervening disease assessment. Two had clinical progression without imaging confirmation.

As expected, myelosuppression was common with grade >3 neutropenia in 42% and grade >3 anemia and thrombocytopenia in 13% (Table 2). Three patients (8.8%) stopped therapy due to myelosuppression. DLT was limited to neutropenia (Table 1). Most patients (79%) stopped therapy due to disease progression. One patient died due to disease progression within 30 days of her last study therapy. There were no treatment‐related deaths.

**TABLE 2:** Treatment‐related adverse events.^a^

	Grade 2, No. (%)	Grade 3, No. (%)	Grade 4, No. (%)
Neutropenia	2 (6)	3 (9)	11 (32)
Neutropenic fever	0	3 (9)	1 (3)
Thrombocytopenia	1 (3)	4 (12)	1 (3)
Anemia	7 (21)	5 (15)	0
Fatigue	8 (24)	1 (3)	0
Mucositis	2 (6)	0	0
Hyponatremia	2 (6)	1 (3)	0
Increased AST	2 (6)	0	0
Insomnia	3 (9)	0	0
Hypokalemia	1 (3)	1 (3)	0
Infection (not neutropenic)	1 (3)	1 (3)	0
Hypocalcemia	1 (3)	1 (3)	0

Abbreviation: AST, aspartate aminotransferase.

^a^ Includes all events with grade 2 or greater toxicity in at least two patients.

**FIGURE 1:**
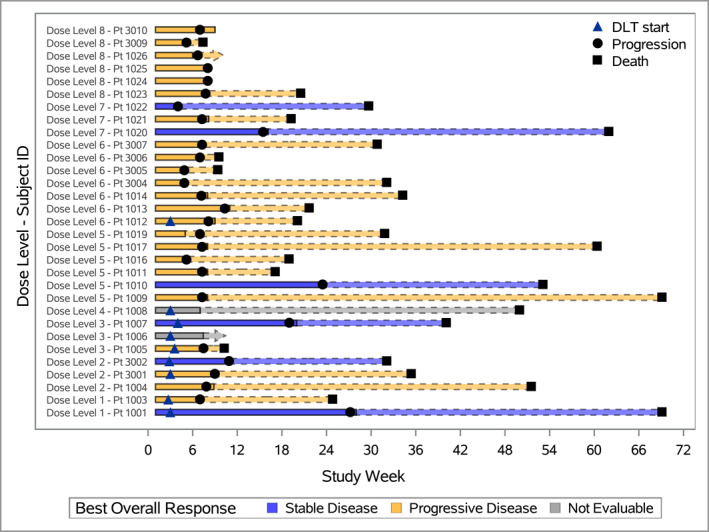
Swimmer plot documenting treatment exposure, DLT, and response (*n* = 31). DLTs are indicated by a triangle, disease progression by a circle, and death a square. The solid outline indicates time on study therapy, whereas the dashed outline denotes time in follow‐up after study therapy was discontinued. Patients with a best response of stable disease are shown in yellow and those with progressive disease in purple. DLT indicates dose‐limiting toxicity.

There were no objective responses and no pt. achieved a clinical benefit response as defined a priori. However, six patients had stable disease, including stability persisting for >4 months in three patients with ER + disease (4.3, 5.4, and 6.3 months) (Figure 1). The patient with stable disease for 6.25 months had a minor response (13% reduction) in the volume of liver metastasis; the other two had predominantly bone and soft tissue disease. Median PFS was 1.7 months (1.6, 1.8), and 6‐month PFS probability was 6.7% (1.2, 19.2). Median overall survival was 7.3 months (4.6, 8.2).

**FIGURE 2:**
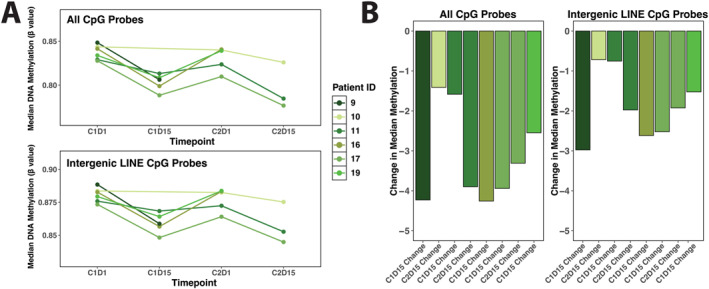
Global loss of DNA methylation in patient PBMCs following treatment. (A) LINE graphs showing the median DNA methylation levels in patient PBMC samples, assessed using all CpG probes or intergenic LINE CpG probes, across treatment time points (*n* = 6). (B) Bar plots depicting the change in median DNA methylation levels in PBMCs 15 days after the start of the treatment cycle (*n* = 8). PBMCs indicates peripheral blood mononuclear cells.

**FIGURE 3:**
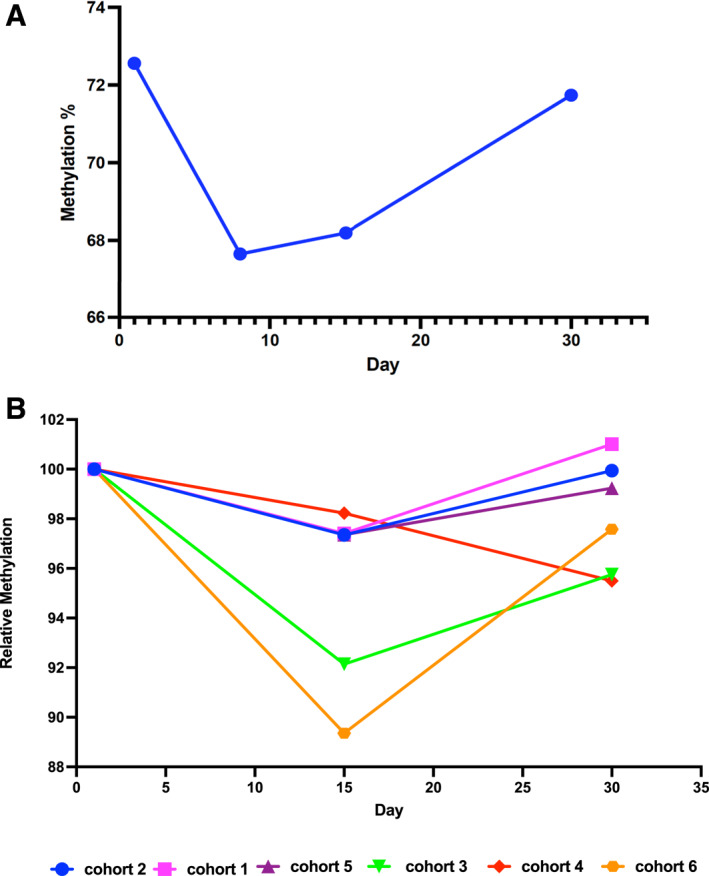
Methylation levels of LINE1 repetitive element. LINE1 was used to assess global DNA methylation changes in PBMCs isolated from (A) baseline and before treatment at different time points in all subjects (*n* = 16), or (B) per cohort (*n* = 16). Changes in methylation were modest and not clearly dose‐related. PBMCs indicates peripheral blood mononuclear cells.

Overall LINE demethylation ranged from 5% to 10%, based on genome‐wide methylation profiling using all CpG probes (Figure 2A, upper), intergenic LINE probes (Figure 2A, lower), and the change in median DNA methylation levels in PBMCs 15 days after the start of the treatment cycle (Figure 2B). Although methylation changes were not dose‐dependent, high‐resolution analysis revealed variable LINE1 demethylation in different doses/cohorts (Figure 3A,B). For example, more pronounced demethylation was observed in two cohorts (Figure 3B, cohorts 3 [green line] and 6 [orange line]). As expected, there was a trend for the sequential regimens to have more demethylation (8.3%) compared to the concurrent dosing cohorts (5.1%), despite a much lower dose of ASTX727 (44 mg/cycle vs. 123 mg/cycle). Although this difference was not significant, the observation provides evidence for an interaction between the two drugs. In the sequential regimen, demethylation seen in cohort 5 (2.6%, *n* = 2, purple line) compared to cohort 6 (10.6% demethylation, *n* = 5, orange line), but the number of patients was small.

INS6, which has a normally highly methylated promoter CpG island, was used to extend demethylation studies from LINE1 to single loci. All 21 cases were examined. INS6 demethylation varied across cohorts, ranging from <1% (e.g., cohort 5) to >11% (e.g., cohort 6) (Figure S1). INS6 demethylation correlated highly with LINE1 (*R* ^2^ = 0.8 for the LINE1 shown in Figure 3). It should be noted that LINE1 is more sensitive to demethylation (demethylates two to three times more than INS6), and no samples showed increased methylation of INS6, confirming assay validity.

**FIGURE 4:**
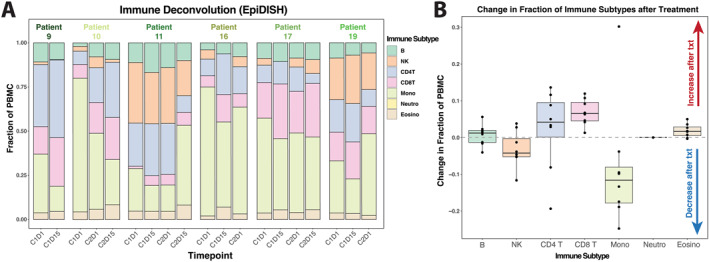
Immune cell dynamics in patient PBMCs following treatment. (A) Immune cell subtype proportions inferred from PBMC samples using EpiDISH deconvolution analysis (*n* = 6). (B) Box plots showing changes in immune cell fractions 15 days after the start of the treatment cycle (*n* = 8). PBMCs indicates peripheral blood mononuclear cells.

Deconvolution analysis of the methylation data demonstrated global and intergenic LINE CpGs changed ∼1%–4% by differential methylation locus analysis at day 15 (Figure 4A,B). Violin plots comparing of β value ranges for CpG site methylation levels pre‐ and posttreatment demonstrated consistent bimodal distributions, with peaks near 0 and 1 corresponding to hypo‐ and hypermethylated regions (Figure S2). There was a general trend (pre‐ vs. posttreatment) toward loss of monocytes and gain of CD8 and CD4 T cells (Figure 4).

## DISCUSSION

A previous phase 1 trial explored the combination of decitabine and talazoparib in patients with AML unfit for or resistant to cytotoxic chemotherapy. That trial enrolled 25 patients over seven cohorts, ultimately arriving at a recommended phase 2 dose of decitabine 20 mg/m^2^ intravenously daily for 5 or 10 days and talazoparib 1 mg orally daily for 28 days. Two patients achieved complete remission with incomplete count recovery, and three patients had hematologic improvement. Pharmacodynamic studies showed the expected DNA demethylation and increased PARP trapping in chromatin. γH2AX foci increased significantly with increasing talazoparib doses combined with 20 mg/m^2^ decitabine.^26^ However, given the nature of AML, hematologic toxicity could not be evaluated in that trial, prompting our first phase 1 trial of a DNMTi with a *PARP*i in patients with solid tumors. The extent of myelosuppression was not predicted by the preclinical models and was even more profound than we had anticipated, leading to multiple amendments to explore alternate dosing strategies.

Although we were able to identify a safe and tolerable dose, real questions remained as to whether the doses were below the level needed for biologic and clinical activity. We did detect changes in methylation in PBMCs, but the changes were small and not clearly dose‐dependent. The correlative analysis is limited by the small samples size and focus on PBMCs. To facilitate rapid accrual, we did not collect serial tumor biopsies so we could not assess changes in methylation in tumor samples. However, work in other settings has found a poor correlation between methylation changes in PBMCs and tumor samples.^33^, ^34^, ^35^, ^36^, ^37^ In addition, it is possible that the hypomethylation effect could be masked by increased cytotoxic activity of talazoparib on demethylated cells. Despite the dose attenuation, we did see some signs of clinical activity in previously treated patients although there were no objective responses, and the duration of stable disease did not meet the a priori definition of clinical benefit. PARP1‐specific inhibitors may have less myelosuppression than the broad inhibitors currently in clinical use.^38^, ^39^, ^40^, ^41^ If synergy is maintained, the combination of a PARP1‐specific inhibitor with a DNMTi may allow greater combined drug exposure and would be worth investigating.

Although we focused on models and patients without BRCA mutations, the synergy may also amplify the benefit of PARPis in patients with deleterious mutation of *BRCA* or other genes in the homologous recombination (HR) pathway.^42^ The combination of ASTX727 and olaparib is being studied in an ongoing phase 1/2 trial (NCT06177171) in patients with advanced/metastatic solid tumors and germline or somatic mutations in genes involved in homologous recombination (i.e., *BRCA1,* *BRCA2, PALB2, ATM,* or *CHEK2*). In addition to defining the maximum tolerated dose and evaluating clinical response, exploratory correlative studies will characterize the various homologous recombination pathway mutations and their functional implications and create patient‐derived xenografts and organoids to study resistance mechanisms.

The synergistic effect could also be maintained by combining PARPis with direct STING agonists.^43^ Pedretti and colleagues^43^ analyzed 35 breast cancer patient‐derived xenografts (PDX) and mouse‐derived allografts (MDA). The cGAS‐STING‐IFN pathways were activated in tumors sensitive to PARPi. The combination of *PARP*i and a novel STING agonist (STINGa) increased immune infiltration and antitumor activity. Notably, additional analyses highlighted the importance of natural killer cell engagement, further supporting combination with immune therapies.^43^

Other mechanisms to expand the potential benefit of PARPi to patients with intact HR have also been studied.^44^, ^45^ The combination of everolimus and niraparib was not feasible, even at reduced doses, due to rapid onset and severe hypertension.^46^ A phase 1b dose escalation and expansion study of the pan‐PI3Ki buparlisib (BKM120) and olaparib found clinical benefit in patients with and without germline *BRCA* mutations but required attenuation of the buparlisib dose due to toxicity.^47^ The α‐specific Pi3K inhibitor alpelisib has also been successfully combined with olaparib. Of 28 patients with epithelial ovarian cancer, 10 (36%) achieved a partial response and 14 (50%) had stable disease; ORR was similar for g*BRCA*mut and g*BRCA*wt patients, 30% and 35%, respectively (Fisher exact test, *p* = .42).^48^ The same olaparib/alpelisib combination was then studied in 17 patients with previously treated TNBC. ORR was 18% (23% for patients treated at the recommended phase 2 dose), and none of the three patients who achieved a partial response had a known BRCA mutation.^49^ The combination of olaparib and the AKT inhibitor capivasertib was well tolerated in a recently reported phase 1 trial. Pharmacodynamic studies confirmed phosphorylated (*p*) GSK3β suppression, increased pERK, and decreased *BRCA1* expression. Antitumor activity was observed in patients harboring tumors with germline BRCA1/2 mutations and BRCA1/2 wild‐type cancers with or without somatic DDR and/or PI3K‐AKT pathway alterations.^50^ Other investigators recently conducted the first study to combine talazoparib with an mTOR/pan‐PI3K inhibitor (gedatolisib) in patients with advanced triple‐negative breast cancer or advanced HER2 negative breast cancer with or without a germline *BRCA1/2* mutation.^51^ The combination was safe but did not meet its primary efficacy response threshold. HRD status (measured by genomic instability) was not correlated with response, suggesting functional HRD assessments may need to be examined.

To better understand the effect of hypomethylating agents on immune cells, we examined DNA methylation changes in patient PBMCs. LINE1 PBMC methylation levels decreased after ASTX727 treatment, and global hypomethylation of CpG loci in PBMCs was also observed. However, an unexpected finding of altered components of PBMCs by ASTX727, including monocytes and lymphocytes, was apparent, and the observed gain in T cells could reflect an altered (activated) tumor microenvironment, one more susceptible to immunotherapy.

This is the first report on the global impact of a hypomethylating agent–PARPi combination treatment on PBMC methylomes in breast cancer patients. The effect of the low‐dose ASTX727 with talazoparib combination recommended for phase 2 trials on PBMC methylation levels was modest, ultimately leaving our underlying hypothesis largely untested in the clinic.

## AUTHOR CONTRIBUTIONS

**Kathy D. Miller**: Conceptualization; data curation; methodology; supervision; project administration; writing—original draft; writing—review and editing; funding acquisition; resources; and investigation. **Alexandra Thomas**: Writing—review and editing; supervision; investigation; and resources. **Sandra Althouse**: Data curation; formal analysis; visualization; and writing—original draft. **Yong Zang**: Data curation; formal analysis; visualization; writing—original draft; and supervision. **Erin Conder**: Data curation; writing—review and editing; project administration; and supervision. **Ryan Burgos**: Writing—review and editing; project administration; supervision; resources; and funding acquisition. **Bryan P. Schneider**: Data curation; investigation; writing—review and editing; and resources. **Tarah Ballinger**: Data curation; writing—review and editing; investigation; and resources. **Emily Douglas**: Data curation; writing—review and editing; and resources. **Katherine Ansley**: Data curation; writing—review and editing; and resources. **H. Josh Jang**: Data curation; formal analysis; visualization; writing—review and editing; and resources. **Woonbok Chung**: Data curation; formal analysis; visualization; writing—review and editing; and resources. **Jean‐Pierre Issa**: Conceptualization; formal analysis; visualization; writing—review and editing; resources; and funding acquisition. **Kenneth P. Nephew**: Conceptualization; formal analysis; visualization; methodology; investigation; supervision; writing—original draft; writing—review and editing; funding acquisition; and resources. **Feyruz V. Rassool**: Conceptualization; formal analysis; visualization; methodology; investigation; supervision; writing—original draft; writing—review and editing; resources; and funding acquisition.

## CONFLICT OF INTEREST STATEMENT

Katherine Ansley reports grand and/or contract funding from AstraZeneca, Daiichi Sankyo, Exact Sciences, Genentech, Gilead Sciences (aka Gilead Foundation), and Pfizer. Tarah Ballinger reports consulting fees from AstraZeneca and Novartis. Emily Douglas reports consulting fees from Novartis. Kathy D. Miller reports participation on a data safety monitoring board for AstraZeneca, Celcuity, F. Hofmann‐La Roche, and Merck. Bryan P. Schneider reports grant and/or contract funding from Genentech, Pfizer, and Foundation Medicine; and participation on a data safety monitoring board or advisory board for Genentech and Eli Lilly. Alexandra Thomas reports consulting fees from Accord, Delphi Diagnostics, Exact Sciences, Medscape, and RTI International; and stock holdings in Bristol‐Myers Squibb, Gilead Sciences Inc, Johnson and Johnson International, and Pfizer International LLC. The other authors disclose no conflicts of interest. Drug supply and partial research support provided by Astex and Pfizer.

## Supporting information

Supplementary Material

Supplementary Material

Supplementary Material

## Data Availability

The data that support the findings of this study are available from the corresponding author on reasonable request.
