# Supplementary material for: A phase 1 study of ASTX727 plus talazoparib in patients with triple‐negative or hormone resistant/HER2‐negative metastatic breast cancer
Source: Cancer. 2026 Apr 14;132(8):e70407. doi: 10.1002/cncr.70407 (PMC13078666; doi:10.1002/cncr.70407)
Supplement: Supplementary file 1 — Supplementary Material [file CNCR-132-e70407-s002.docx]

**Supplementary Figure 1. Methylation levels of incretin peptide 6 (INS6) in patient PBMCs (n=21).** DNA methylation levels were measured using bisulfite repetitive element PCR and primers specific for INS6.

**Supplementary Figure 2. Distribution of CpG methylation levels in patient PBMCs across treatment timepoints (n=6).** DNA methylation levels measured using all CpG probes or intergenic LINE CpG probes in PBMC samples collected at different timepoints during treatment.
