# Supplementary figures and images for: A phase 1 study of ASTX727 plus talazoparib in patients with triple‐negative or hormone resistant/HER2‐negative metastatic breast cancer
Source: Cancer. 2026 Apr 14;132(8):e70407. doi: 10.1002/cncr.70407 (PMC13078666; doi:10.1002/cncr.70407)

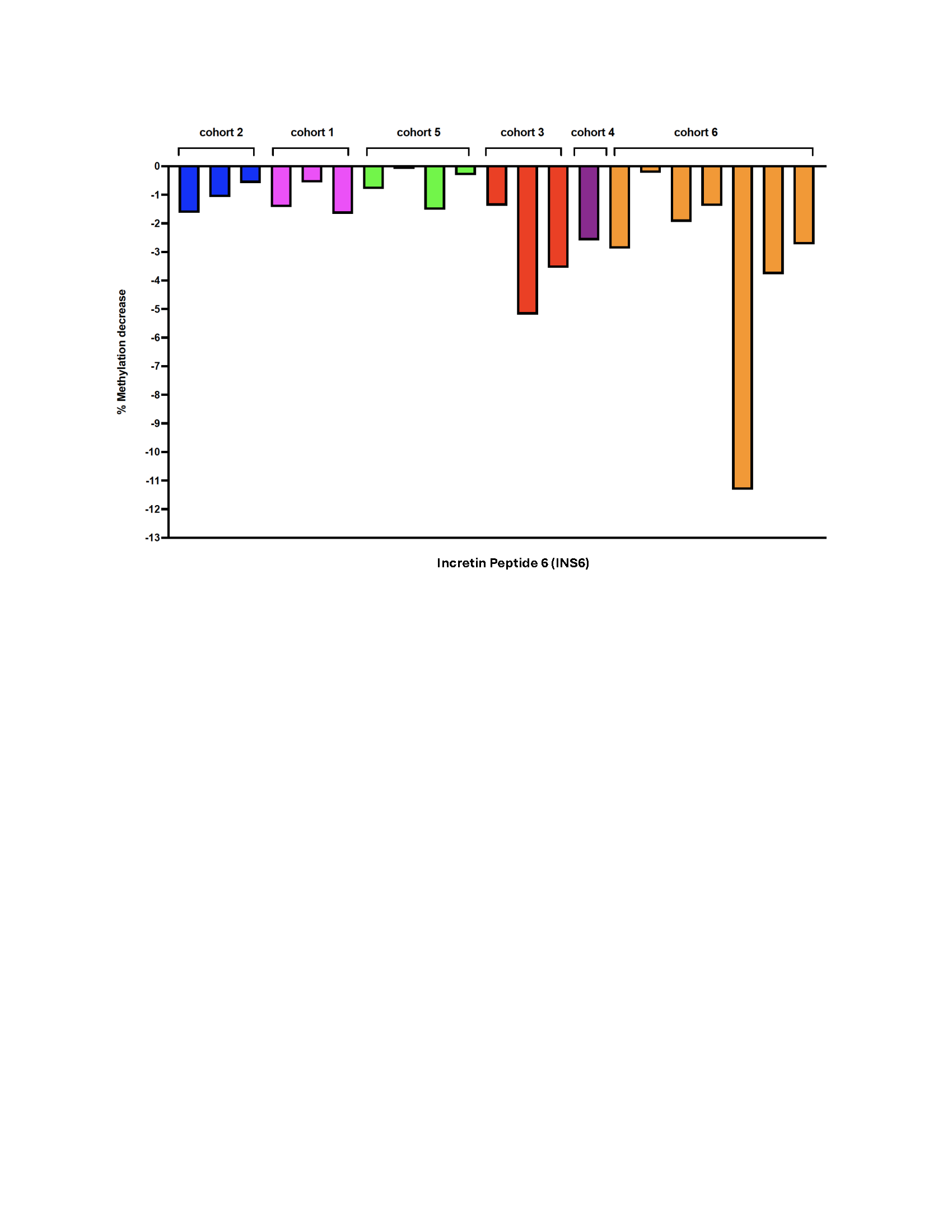

Supplement: Supplementary file 2 — Supplementary Material [file CNCR-132-e70407-s001.tif]

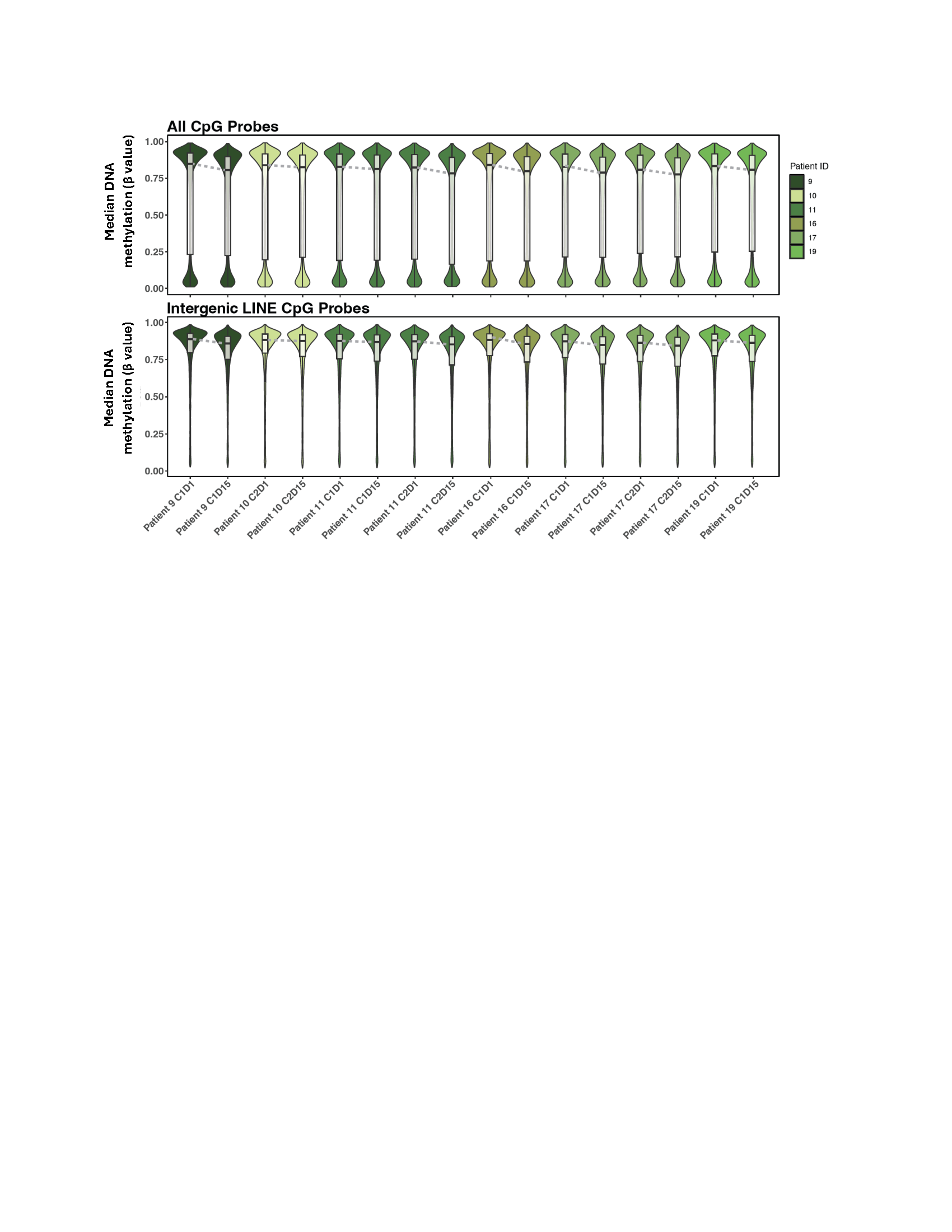

Supplement: Supplementary file 3 — Supplementary Material [file CNCR-132-e70407-s003.tif]
